# Surgical management of chronic calcific deposit resembling tendon sheath infection in the index finger: A rare case report and literature review

**DOI:** 10.3389/fsurg.2023.1119612

**Published:** 2023-04-19

**Authors:** Deming Guo, Jingjing Yang, Xiaoning Liu

**Affiliations:** ^1^ Orthopaedic Medical Center, Second Hospital of Jilin University, Changchun, China; ^2^ Operating Room, Second Hospital of Jilin University, Changchun, China

**Keywords:** calcific tendonitis, distal interphalangeal joint, finger, surgical treatment, case report

## Abstract

**Background:**

Calcific tendonitis rarely occurs in the fingers, and it is easily misdiagnosed. Herein we describe the case of a patient with multiple calcific lesions within the flexor digitorum superficialis and the extensor digitorum tendons of the distal interphalangeal joints of the right index finger, and the surgical treatment of those lesions.

**Case presentation:**

The patient was a 66-year-old man who reported pain and swelling in his right index finger for one year. He was diagnosed with chronic calcific tendonitis based on his symptoms and radiology images. He was successfully treated surgically, and histopathological examination confirmed the diagnosis. After one month, the patient had healed well, and there was no recurrence.

**Conclusions:**

This is the first report of a patient suffering from chronic calcific tendonitis in a finger who failed conservative treatment and was successfully treated with surgery. The outcome demonstrates that surgical debridement can yield a good outcome in patients with chronic calcific tendonitis.

## Introduction

Calcific tendinitis is an acute idiopathic inflammatory disease that involves pain, tenderness, and edema (1). It mainly occurs in joints or tendons, and most commonly occurs in shoulder joints. It only occurs in the hands in approximately 2.4% of cases (2). Calcific tendinitis in the fingers has rarely been reported. Due to its low incidence and atypical clinical manifestations, calcific tendonitis in the fingers is usually misdiagnosed as flexor sheath infection, tenosynovitis, septic arthritis, fracture, cellulitis, or gout (3–5). Such misdiagnoses have resulted in unnecessary antibiotic treatment and surgery. Conservative treatment is usually administered for calcified tendinitis, including splinting and non-steroidal anti-inflammatory medications (6). The effects of conservative treatment can be insufficient however, and the condition is prone to relapse. When conservative treatment is ineffective, or the symptoms persist, surgery can be considered (5, 6).

Herein we describe a case of chronic calcified tendinitis in the distal interphalangeal (DIP) joint of the right index finger. The calcifications were located in both the flexor digitorum superficialis (FDS) and the extensor digitorum tendons of the right index DIP joint. Surgery was performed, and complete finger photography, x-rays, and histopathological examinations were conducted before and after surgery.

This report is intended to provide orthopedic surgeons with a better understanding of calcific tendinitis, particularly with respect to surgical outcomes and specific treatment concerns. The patient provided informed written consent for the publication of the report, including all images, and the Jilin University Second Hospital Institutional Review Board approved the publication of the report.

## Case presentation

**Figure 1:**
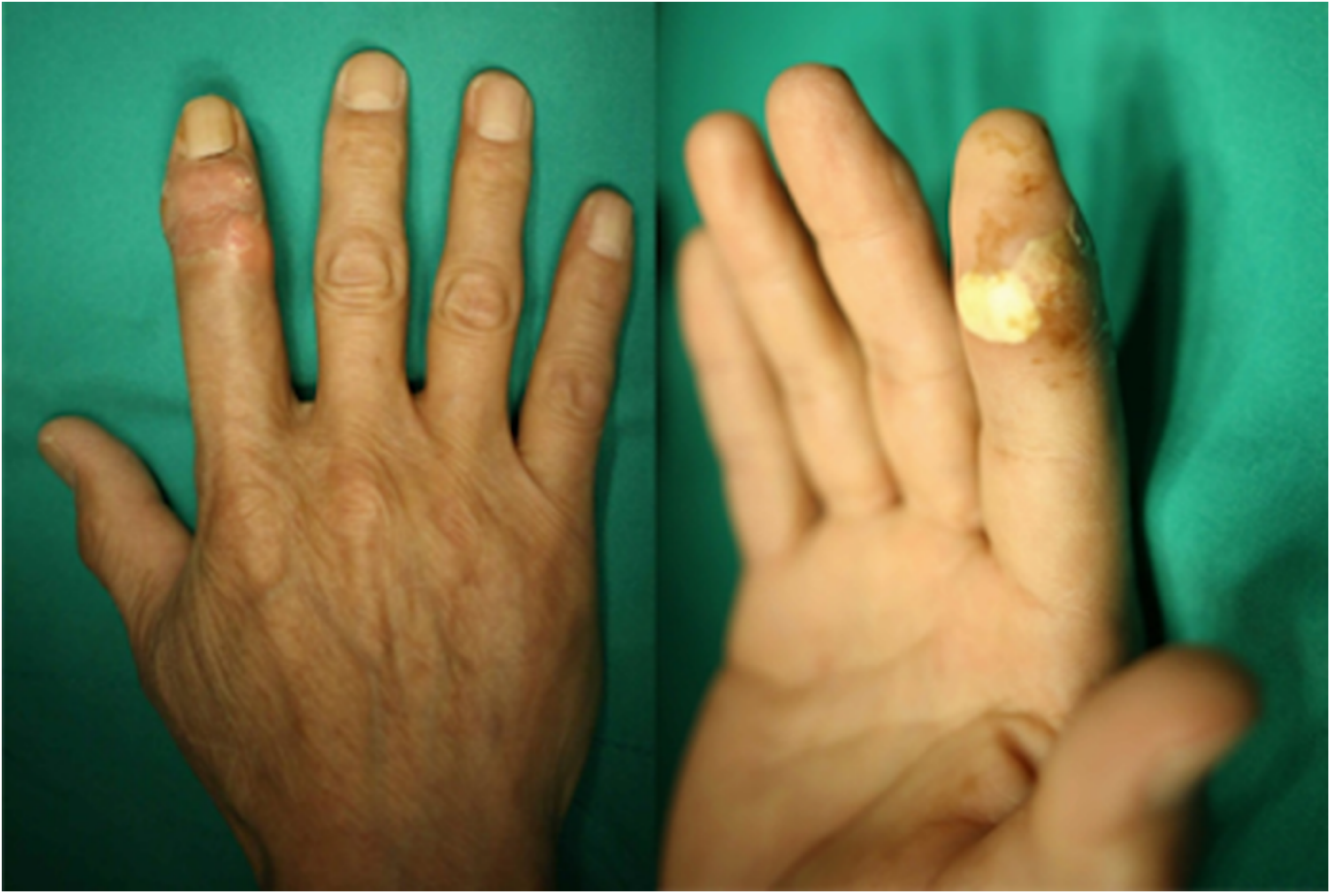
The right index finger with slight swelling and redness on the back of the right index distal interphalangeal joint, and obvious swelling and paleness on the palm side in the distal interphalangeal joint.

**Figure 2:**
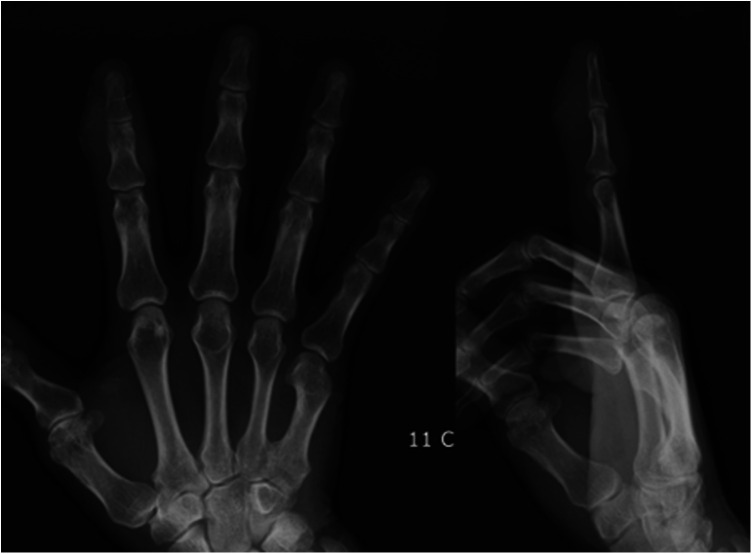
Preoperative x-ray of the right index finger depicting soft tissue swelling at the distal interphalangeal joint.

A 66-year-old man presented with pain and swelling of the right index finger. He reported that he had had it for one year, which had progressively worsened during the previous week. There was no history of trauma, fever, injury, or similar episodes of pain. The results of serological examinations were normal. Physical examination revealed slight swelling, redness, and tenderness on the back of the right index DIP joint, and noticeable swelling, paleness, and tenderness on the palm side (Figure 1). The patient was initially diagnosed with chronic tendon sheath infection, but there were no typical Kanavel symptoms, such as tenderness along the flexor sheath, fusiform swelling, or pain on passive extension or with the finger held in partial flexion (7). x-rays of the right index finger depicted soft tissue swelling at the DIP joint (Figure 2). Collectively these results suggested calcific tendonitis in the FDS and extensor digitorum tendons of the DIP joint.

**Figure 3:**
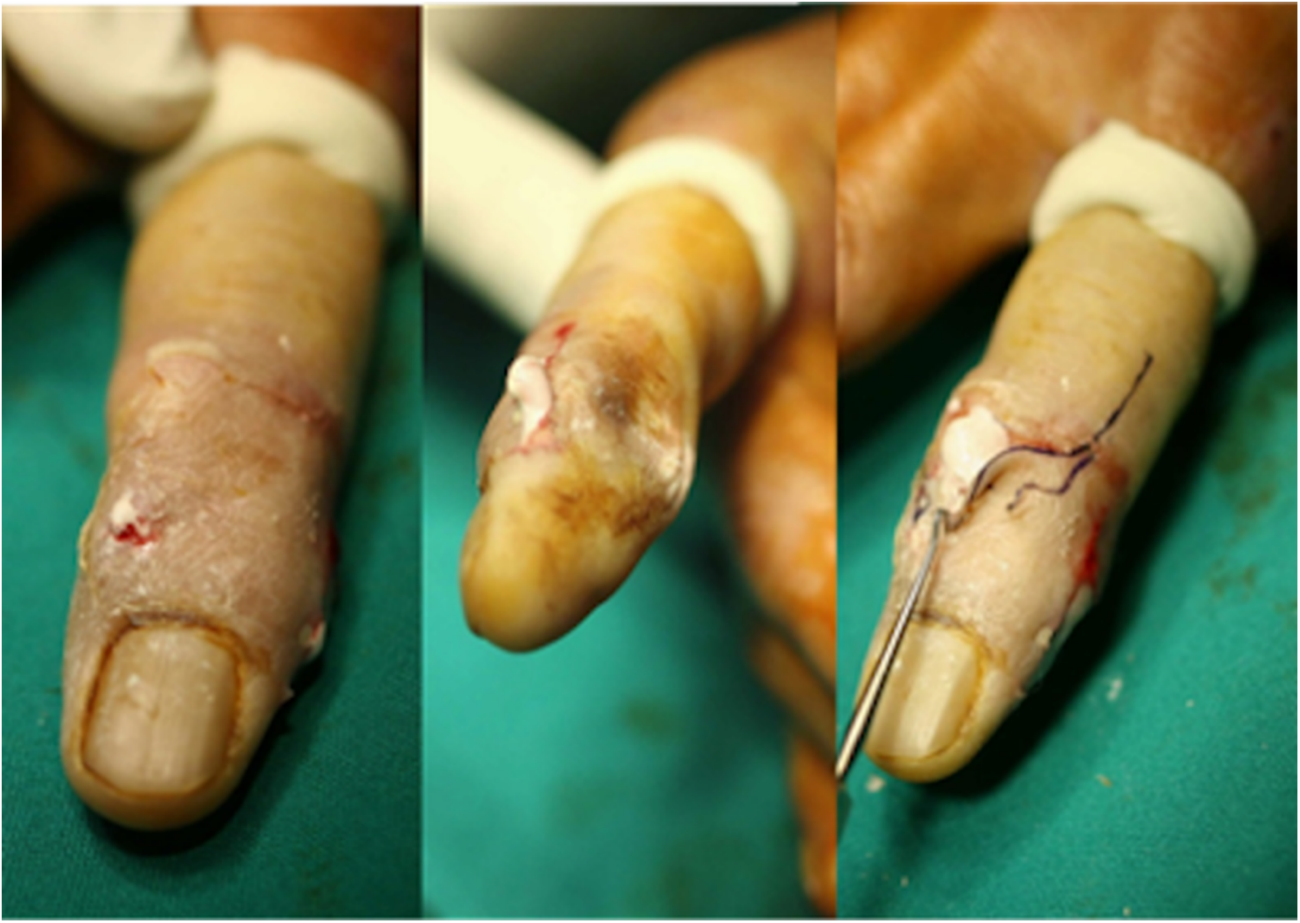
The calcifications during the operation.

Due to the long period of disease and severe clinical manifestations, surgical treatment was recommended to the patient. He opted to take the surgery, and the operation was performed under local anesthesia. The patient was placed in a supine position with his right hand on the operating table. After a temporary rubber ring tourniquet was placed behind the metacarpophalangeal (MCP) joint, a zigzag incision was made on the back of the DIP joint. The ulnar side was then incised to the level of the FDS tendon of the index finger, and small calcifications were detected (Figure 3). A 1 cm × 1 cm calcification was detected *via* radial exploration of the extensor digitorum tendon on the palm of the finger. All of the calcifications were removed without tendon damage. The resected tissue was sent for histological examination.

**Figure 4:**
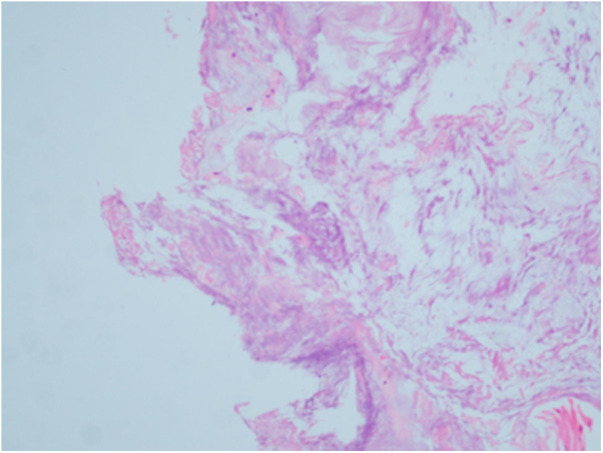
Postoperative histopathological examination revealed calcium hydroxyapatite deposition with necrosis of surrounding tissue.

**Figure 5:**
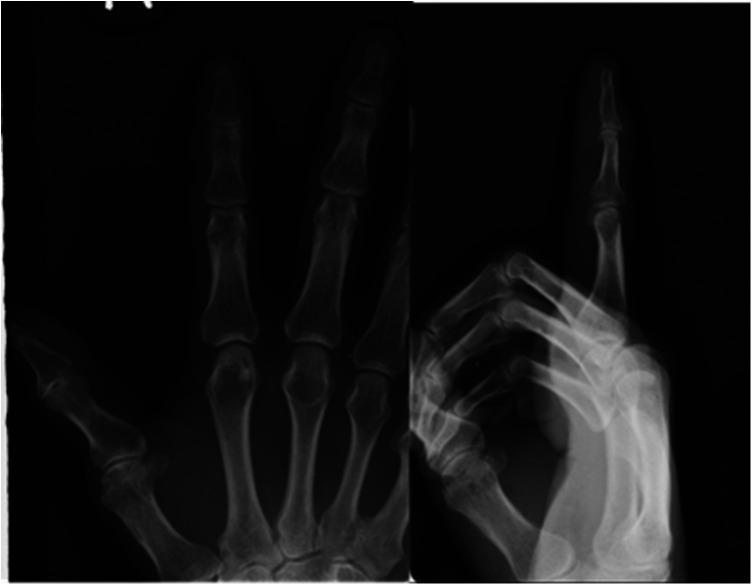
Postoperative x-ray depicting no residual calcifications.

**Figure 6:**
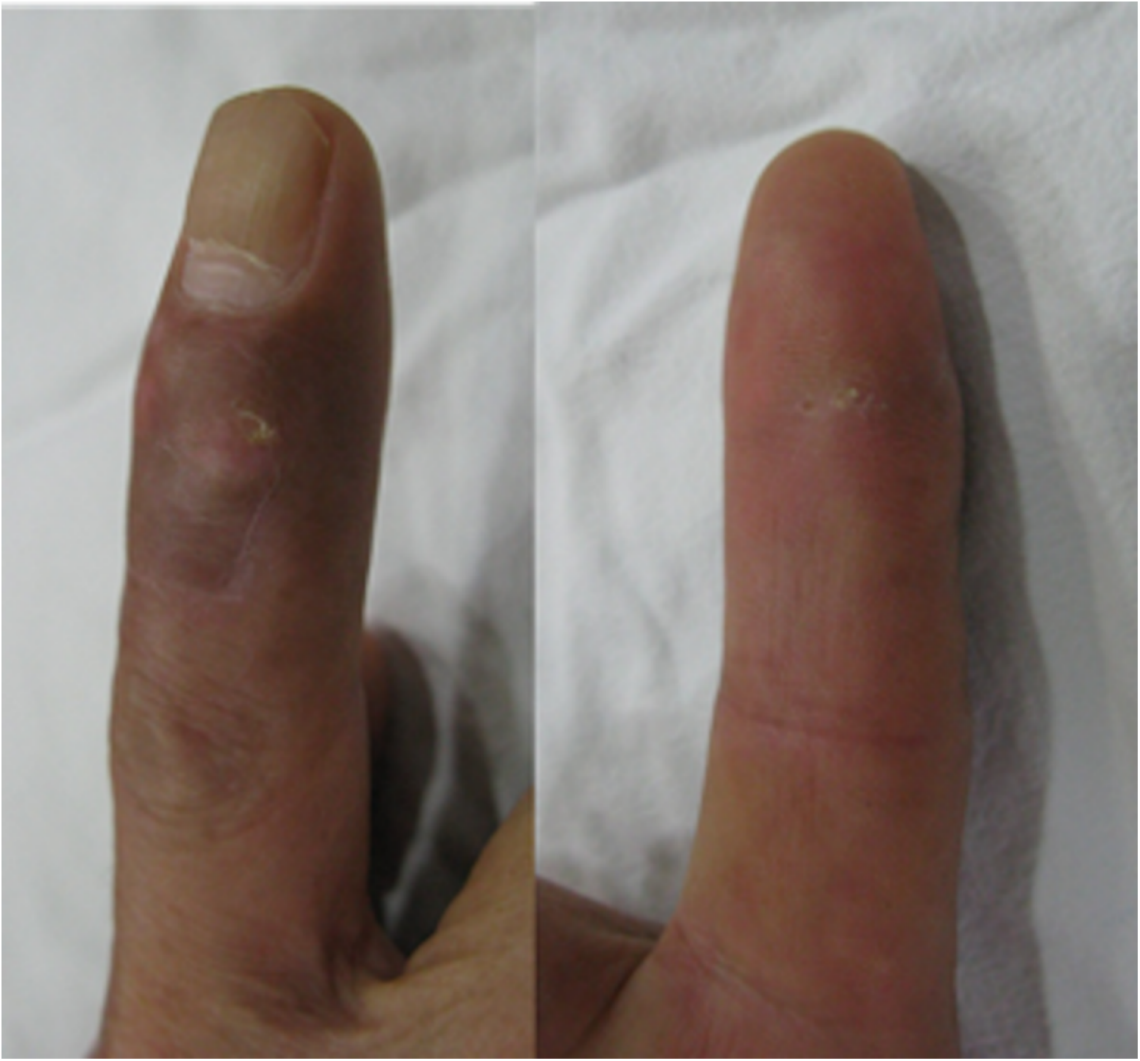
The finger after 1 month, by which time the pain and swelling had completely subsided.

Hematoxylin-eosin staining of histological sections of the resected tissue revealed calcium hydroxyapatite deposition with necrosis of surrounding tissue (Figure 4), confirming the diagnosis of calcific tendinitis. Postoperative x-rays did not depict any residual calcification. One month after surgery, the pain and swelling had completely disappeared (Figures 5, 6).

## Discussion and conclusions

Calcific tendinitis is caused by hydroxyapatite crystals or carbonate apatite, but its precise etiology is unknown (1). Some scholars believe that it is caused by localized tissue hypoxia or trauma (8, 9), and that hypoxia of the tendon is an inductive event. Poor blood perfusion caused by mechanical or metabolic problems causes the tendon to be converted into fibrocartilage, and chondrocytes mediate calcium salt deposition. The main factors influencing calcific tendinitis are female sex, trauma to the tendon causing local hypoxia (including repetitive trauma), autoimmune diseases such as rheumatoid arthritis, and metabolic conditions including diabetes (5, 10).

The main clinical manifestations of calcific tendinitis include acute erythema, swelling of the lesion, and limited activity (1). Rather than the calcification itself, most symptoms of calcific tendinitis result from the destruction of surrounding soft tissues caused by the calcified foci, which leads to non-specific inflammatory reactions (10). Therefore, the clinical symptoms of calcific tendinitis are not typical. Results of serological examinations are usually normal, including serum calcium, phosphorous, alkaline phosphate, and uric acid levels (11, 12). Occasionally the white blood cell count and the erythrocyte sedimentation rate are elevated. Accordingly, a serological examination can be useful for distinguishing calcific tendinitis from tendon sheath infection and gout, but not from a fracture (6). As in the present case, radiology imaging depictions of calcific tendinitis can be relatively mild, and this can be used to distinguish calcific tendinitis from a fracture (13–15). It is these inconspicuous clinical examination features that can render the diagnosis of calcific tendinitis difficult.

Calcific tendinitis most commonly occurs in the shoulder joint, followed by the knees and hips, and it rarely occurs in the hands or wrists. In the hands, the most common location is the carpal tunnel (16). The most common location of calcific tendinitis occurring in the finger is the MCP joint, followed by the proximal interphalangeal joint, and the least common is the DIP joint (17). To our knowledge, a total of 26 cases of calcific tendinitis in the fingers have previously been reported (Table 1). The most common joint involved has been the MCP, and the most common digit involved has been the thumb (Table 2).

**Table 1:** Summary of the literature.

Study	Sex	Age (years)	History of symptoms	Location				Treatment	Treatment period	Follow-up
				Hand	Finger	Joint	Tendon			
Ali and Kelly (2004) (18)	M	66	4 days	Lt.	3rd	PIP	FDS	NSAIDs	10 days	–
Doumas et al. (2007) (19)	F	44	5 days	Lt.	1st	IP	–	splinting and NSAIDs	10 days	–
	M	47	3 days	Lt.	3rd	MCP	–	splinting and NSAIDs	1 week	–
Hakozaki et al. (2007) (20)	M	10	1 day	Lt.	1st	IP	EPL	sprinting and NSAIDs	1 week	6 weeks
Shields et al. (2007) (21)	F	mid-20s	11 days	Lt.	1st	MCP	RCL	local injection, splinting, and NSAIDs	5 days	10 months
	M	late 20s	14 months	Rt.	1st	MCP	RCL	local injection, splinting, and NSAIDs	–	Relapse after 6 months, no relapse after 6 months with original treatment plan
Wiper and Garrido (2008) (22)	F	56	2 months	Rt.	2nd	MCP	FDS	NSAIDs	–	6 weeks
Lee et al. (2012) (23)	M	34	2 weeks	Lt.	1st	MCP	APB	NSAIDs	2 weeks	–
Munjal et al. (2013) (24)	M	51	2 days	Rt.	2nd	DIP	FDS	splinting and NSAIDs	5 days	3 weeks
Kim et al. (2014) (25)	F	22	1 week	Lt.	4th	PIP	FDS	splinting and NSAIDs	2 weeks	6 months
Kim et al. (2016) (26)	F	72	3 days	Lt.	4th	DIP	RCL	splinting and NSAIDs	1 week	2 months
Walocko et al. (2017) (27)	M	9	2 days	Lt.	2nd	PIP	FDS	splinting and NSAIDs	3 weeks	–
Tomouk and Birks (2019) (28)	F	middle age	2 days	Lt.	4th	DIP	FDS	Conservation treatment	–	–
Tsoi and Hughes (2019) (29)	F	30	1 year	Rt.	3rd	MCP	RCL	local injection, splinting and NSAIDs	4 weeks	2 months
Zribi et al. (2019) (12)	M	36	chronic pain	–	1st	MCP	LCL	splinting, analgesics, NSAIDs, and icing	3 weeks	
Kim et al. (2018) (6)	F	54	–	Rt.	2nd	MCP	–	warm baths, NSAIDs, and splinting	–	6 months
				Lt.	1st	MCP	––			
	F	64	–	Rt.	1st	IP	–		–	9 months
				Lt.	5th	PIP	–			
	F	52	–	Rt.	2nd	MCP	–		–	6 months
	F	39	–	Rt.	3rd	MCP	–		–	9 months
				B/L	5th	MCP	–			
	F	58	–	Lt.	1st	MCP	–		–	9 months
				B/L	5th	MCP	–			
	F	46	–	Rt.	3rd	PIP	–		–	9 months
	M	32	–	Lt.	1st	MCP	–		–	12 months
				Lt.	2nd	PIP	–			
	F	52	–	Rt.	3rd	PIP	–		–	9 months
	F	46	–	Rt.	3rd	PIP	–		–	18 months
	F	51		Lt.	2nd	PIP	–		–	24 months
Current study	M	66	1 year	Rt.	2nd	DIP	both EPL and FDS	surgery	–	1 month

M, male; F, female; Rt., right; Lt., left; B/L, bilateral; MCP, metacarpophalangeal; PIP, proximal interphalangeal; IP, interphalangeal; DIP, distal interphalangeal; NSAIDs, nonsteroidal anti-inflammatory drugs; RCL, radial collateral ligament; FDS, flexor digitorum superficialis; LCL, lateral collateral ligament; EPL, extensor pollicis longus; APB, abductor pollicis brevis.

**Table 2:** Digit and joint level locations of 26 previously reported calcific tendinitis lesions.

	Location	Number	%
Joint level	MCP	14	42.4
	PIP	12	36.4
	DIP	4	12.1
	Thumb IP	3	9.1
Digit	Thumb	10	30.3
	Index finger	9	27.3
	Long finger	6	18.2
	Ring finger	5	15.2
	Little finger	3	9.1

MCP, metacarpophalangeal; PIP, proximal interphalangeal; DIP, distal interphalangeal; IP, interphalangeal.

Calcific tendinitis is a self-limiting disease, but both conservative treatment and surgical treatment have been successfully utilized (5, 30). Conventional treatment methods involve immobilization in conjunction with aspirin or non-steroidal anti-inflammatory medications, with the aim of reducing pain and relieving symptoms. These measures can be tried for at least six months before considering surgical treatment, particularly in cases involving a finger (6). Surgical intervention is more suitable in patients with severe symptoms and, or a long period of disease, or after the failure of conservative treatment (31).

All the patients in the recently reported cases have opted for conservative treatment (6, 13, 18–20, 22–29). The characteristics of these patients included common clinical symptoms and mild medical histories. During the treatment follow-up periods, only one patient exhibited a recurrence of calcified deposits within 18 months (21). Calcium deposits on the hands are highly misdiagnosed not only because of their similar symptomology to more common conditions, but also because clinicians are unfamiliar with the disease process. The differential diagnosis is broad and includes infections, fractures, metabolic disorders, degenerative and autoimmune diseases (17, 27). Due to the severe symptoms in the present case, however, as well as the long disease course and the associated substantial effects on quality of life, the adequacy of conservative treatment could not be predicted. It is also notable that the operation is simple and easy, they can yield curative effects rapidly. Therefore, surgery was recommended to the patient in the current case.

In conclusion, this is the first case report of a patient suffering from multiple chronic calcific tendonitis lesions in the finger who failed conservative treatment and was successfully treated with surgery. The present case demonstrates that surgical repair can yield a good outcome in patients with chronic calcific tendonitis. Orthopedic surgeons should consider the possibility of calcified tendinitis when they encounter patients with hand swelling and pain. Although some calcific tendinitis patients can be cured *via* conservative treatment, we recommend surgical intervention in patients with severe symptoms. It is critical for surgeons to evaluate the disease status of patients with calcific tendinitis in a finger comprehensively and develop the most suitable treatment plan.

## Data Availability

The raw data supporting the conclusions of this article will be made available by the authors, without undue reservation.
